# Versatile metal-wire waveguides for broadband terahertz signal processing and multiplexing

**DOI:** 10.1038/s41467-022-27993-7

**Published:** 2022-02-08

**Authors:** Junliang Dong, Alessandro Tomasino, Giacomo Balistreri, Pei You, Anton Vorobiov, Étienne Charette, Boris Le Drogoff, Mohamed Chaker, Aycan Yurtsever, Salvatore Stivala, Maria A. Vincenti, Costantino De Angelis, Detlef Kip, José Azaña, Roberto Morandotti

**Affiliations:** 1 Institut national de la recherche scientifique, Centre Énergie Matériaux Télécommunications, Varennes, QC J3X 1P7 Canada; 2 Department of Engineering, University of Palermo, Viale delle Scienze, Palermo, 90128 Italy; 3 Faculty of Electrical Engineering, Helmut Schmidt University, Holstenhofweg 85, Hamburg, 22043 Germany; 4 Department of Information Engineering, University of Brescia, Via Branze 38, Brescia, 25123 Italy

**Keywords:** Terahertz optics, Photonic devices

## Abstract

Waveguides play a pivotal role in the full deployment of terahertz communication systems. Besides signal transporting, innovative terahertz waveguides are required to provide versatile signal-processing functionalities. Despite fundamental components, such as Bragg gratings, have been recently realized, they typically rely on complex hybridization, in turn making it extremely challenging to go beyond the most elementary functions. Here, we propose a universal approach, in which multiscale-structured Bragg gratings can be directly etched on metal-wires. Such an approach, in combination with diverse waveguide designs, allows for the realization of a unique platform with remarkable structural simplicity, yet featuring unprecedented signal-processing capabilities. As an example, we introduce a four-wire waveguide geometry, amenable to support the low-loss and low-dispersion propagation of polarization-division multiplexed terahertz signals. Furthermore, by engraving on the wires judiciously designed Bragg gratings based on multiscale structures, it is possible to independently manipulate two polarization-division multiplexed terahertz signals. This platform opens up new exciting perspectives for exploiting the polarization degree of freedom and ultimately boosting the capacity and spectral efficiency of future terahertz networks.

## Abstract (web summary)

Waveguides that can provide complex signal-processing functionalities while guiding terahertz signals are desired. Here, the authors report the independent processing of multiplexed signals by engineering the metal surface of a four-wire waveguide.

## Introduction

Driven by the global demand for high-data-rate communication links, the use of terahertz (THz) radiation^1^ (with frequencies spanning the range between 0.1 and 10 THz) to carry data-streams will soon become unavoidable^2^. Despite the recent dramatic push towards THz wireless communications^3–5^, THz waveguides serve as an indispensable alternative when a free-space propagation approach is unfeasible^6–8^. In general, THz waveguides that can preserve both the broad bandwidth and low dispersion of a free-space link are desirable. Metal waveguides that support transverse electromagnetic (TEM) modes, such as parallel-plate waveguides^9^ (PPWGs) and two-wire waveguides^10^ (TWWGs), are promising candidates due to their capabilities to support the low-loss and low-dispersion propagation of broadband THz pulses. In particular, compared to the large footprints of PPWGs, TWWGs have several distinct advantages, including structural simplicity^11^, tolerance to bending^12^, and affinity to cables for efficient and straightforward connections^13^. Besides the efficient guiding of broadband THz pulses, these waveguides are expected to provide a number of options for versatile signal processing^14,15^. However, in contrast to the recent advances in PPWG-based signal-processing devices, such as power splitters^16^, multiplexers/demultiplexers^17,18^, and add-drop filters^19^, few signal-processing functionalities have been realized in TWWGs. The underlying reason is that the modal energy is tightly confined in the wavelength-scale space between the two wires^10^, which limits the possible ways to manipulate the propagating THz waves. While Bragg gratings, considered among the most fundamental processing units, have been realized in TWWGs, they typically rely on a hybrid approach that requires inserting standalone metalized papers^20,21^ or dielectric gratings^22^ into the waveguides. This, in turn, severely hampers the realization of more sophisticated devices and complex processing functionalities.

Here, we introduce a universal approach for the realization of broadband THz signal processing in metal-wire waveguides by engineering the wire surfaces, in turn leading to the realization of Bragg gratings directly on the metal-wires without the need of introducing additional materials. This is because the THz guidance in metal-wire waveguides is based on the propagation of THz surface plasmon polaritons (SPPs) along the metal-air interface, which is extremely sensitive to the metal surface conditions. Such an approach can be incorporated into innovative waveguide designs, further allowing for the realization of more complex signal-processing functionalities. As a proof-of-concept, we propose a versatile metal-wire waveguide topology, namely a four-wire waveguide (FWWG), which is capable of sustaining two independent and orthogonally polarized fundamental modes, thus acting as a broadband polarization-division multiplexer. By integrating Bragg gratings on metal-wires into such a component, we demonstrate the independent manipulation of polarization-division multiplexed THz signals. The proposed device enables polarization-division multiplexing in waveguides with remarkable structural simplicity, while featuring unprecedented capabilities towards processing multiplexed signals over a broad THz frequency range.

## Results

### Directly engraving of multiscale-structured Bragg gratings on metal-wires

**Fig. 1:**
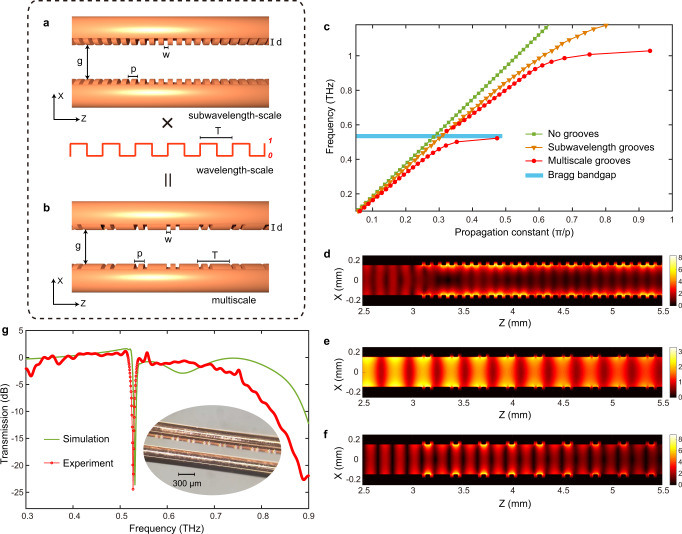
Direct engraving of Bragg gratings on metal-wires. Based on a two-wire waveguide (TWWG) geometry (with a wire radius *r* = 127 µm and an air gap *g* = 300 µm), Bragg gratings are realized by directly engraving grooves with multiscale structures (150-periods-long) along the metal-wires. **a** Schematic of the TWWG with sub-*λ* periodic grooves. Geometry of the grooves: width *w* = 35 µm, depth *d* = 40 µm, and period *p* = 80 µm. **b** Schematic of the TWWG with multiscale grooves. This structure is achieved by superimposing a wavelength-scale periodic modulation with period *T* = 280 µm onto the sub-λ periodic grooves shown in (**a**). **c** Simulated dispersion relations for the plain TWWG (no grooves) and the TWWGs with sub-*λ* and multiscale grooves. **d** Simulated electric field modulus distributions, evaluated at 1.2 THz, in the TWWG with sub-*λ* grooves, showing that the THz energy is mainly trapped within the grooves, preventing further propagation. Simulated electric field modulus distributions in the TWWG with multiscale grooves evaluated at 0.53 THz (**e**) and at 1.0 THz (**f**), respectively. **g** Simulated and experimental transmission spectra of the TWWG with the integrated Bragg gratings. The experimental result is obtained via a standard THz time-domain spectroscopy (TDS) system. The transmission spectra are achieved by calculating the ratio between the power spectra of the signals propagating through the TWWGs with and without grooves. The inset shows the optical microscopic image of the Bragg gratings directly etched on one of the two wires.

When a periodic array of grooves is engraved on the metal surface, surface states behaving like SPPs, so-called spoof SPPs, can be supported^23^. Depending on the spatial period, a subwavelength-scale (sub-*λ*) periodic structure can be treated as an effective medium, while a wavelength-scale periodic structure behaves as a Bragg grating^24^. Figure 1a shows a sub-*λ* periodic array of grooves in a TWWG. The dispersion relation of such a structure (Fig. 1c) is not linear and the group velocity *v*_g_ of the spoof SPPs decreases as the frequency increases. In addition, a cut-off frequency *f*_c_ occurs at ~1.2 THz, indicating that the spoof SPPs at such a frequency are inhibited^25^ and the frequencies above *f*_c_ cannot be guided (Fig. 1d). The propagation characteristics (i.e., *v*_g_ and *f*_c_) of the spoof SPPs can be tailored by engineering the depth of the grooves *d* and the duty cycle *w/p*^25^ (see Supplementary Note 1). When the geometry of the grooves is adjusted to the wavelength scale, *f*_c_ will accordingly shift to a lower frequency range, in turn narrowing the operating bandwidth of the waveguide. In addition, from a practical viewpoint, fabricating trenches with a wavelength-scale geometry on metal-wires is simply not feasible, since it would greatly lower the robustness of the wires, thus potentially leading to their fracture when tension is applied. More importantly, by engraving grooves with a single periodicity on the metal-wires, it is impossible to achieve a Bragg resonance spanning the operating THz bandwidth.

To overcome this issue, while maintaining the operating bandwidth and the wire robustness, we introduce in the THz domain the concept of multiscale structures (Fig. 1b), realized by superimposing a wavelength-scale periodic modulation of *T* = 280 µm onto the sub-*λ* periodic grooves (see Supplementary Note 1). The dispersion relation of the multiscale grooves (Fig. 1c) indicates the existence of a Bragg bandgap at 0.53 THz, given by *f*_Bragg_ = *c*/(2*T*)^26^, where *c* is the speed of light. The THz electric field distributions also confirm the occurrence of a notch frequency at 0.53 THz (Fig. 1e), consistent with the Bragg condition, and the local trapping of the THz energy at 1.0 THz (Fig. 1f). We fabricate a TWWG with integrated Bragg gratings (see Methods) by directly engraving the multiscale grooves along one of the two wires. As shown in Fig. 1g, the experimental transmission spectrum exhibits a Bragg resonance at ~0.53 THz with a notch depth of ~25 dB, which matches the simulated transmission spectrum quite well. The achieved linewidth of such a Bragg resonance (with a *Q*-factor of ~479.5) is remarkably narrow, due to the application of a large number of periods (150 periods). By varying the wavelength-scale modulation *T*, the location of the Bragg resonance can be easily tuned over a broad frequency range within 1 THz.

### Polarization-division multiplexing of THz pulses within FWWGs

**Fig. 2:**
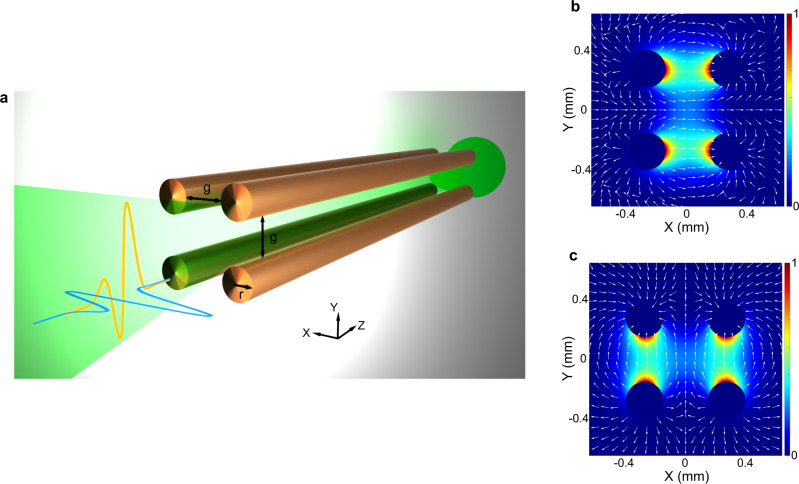
Geometry and fundamental TEM modes of the four-wire waveguide (FWWG). **a** Schematic of the FWWG architecture. The FWWG consists of four identical copper wires (radius *r* = 127 µm) equally separated by an air gap (*g* = 300 µm), arranged into a square geometry. **b**, **c** Simulated electric field intensity distributions of the fundamental TEM modes evaluated at 0.5 THz, TEM_*x*_ (**b**), and TEM_*y*_ (**c**), which can be efficiently excited by *x*-polarized and *y*-polarized THz beams, respectively. The arrows in the 2D distributions indicate the local electric field polarization directions.

Building on the concept of TWWG, we introduce the FWWG platform, which features excellent capabilities in terms of signal transporting (see Supplementary Note 2). The FWWG consists of four identical copper wires (radius *r* = 127 µm) equally separated by an air gap (*g* = 300 µm), arranged into a square geometry, as depicted in Fig. 2a. Its two fundamental modes, TEM_*x*_ (Fig. 2b) and TEM_*y*_ (Fig. 2c), exhibit symmetric field profiles, which are equally divided into two identical portions along the axes. Notably, each portion of the field distribution is mainly confined in-between the two corresponding wires and shows a similar profile to that of the TWWG, thus indicating that the FWWG can also be efficiently accessed by a linearly polarized THz beam. Specifically, TEM_*x*_ is excited within the FWWG by an *x*-polarized THz beam, while TEM_*y*_ is excited by a *y*-polarized THz beam. Remarkably, the FWWG is capable of guiding linearly-polarized THz signals with arbitrary polarization directions. This is because an arbitrary linearly-polarized THz beam coupled into the FWWG is decomposed into the two orthogonal polarization states, which then propagate independently in terms of its fundamental TEM modes. Experimental results detailed in Supplementary Note 2 extensively prove the ability of the FWWG to couple and transmit broadband THz pulses with arbitrary linear polarization directions in a low-loss and low-dispersion manner. It is worth mentioning that, in contrast to all the other existing THz waveguides^27^, this is a unique characteristic of the FWWG.

**Fig. 3:**
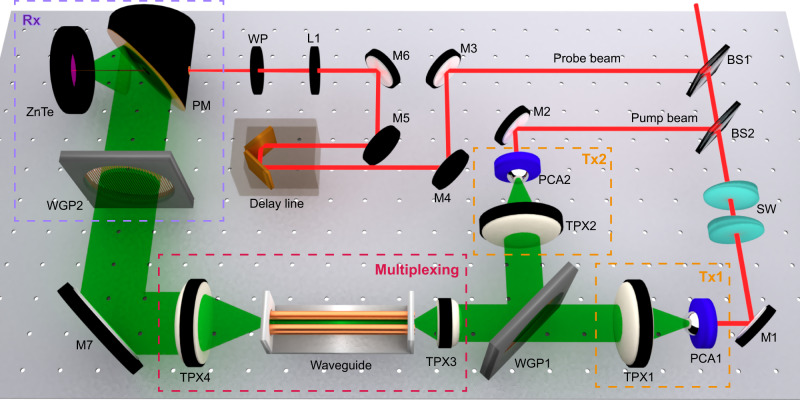
Schematic of the experimental setup. For the demonstration of polarization-division multiplexing within the FWWG, two independent broadband transmitters (Tx1 and Tx2) and a single receiver (Rx) are employed. Two identical photoconductive antennas (PCAs) serve as transmitters, emitting a *x*-polarized THz beam in Tx1 and a *y*-polarized THz beam in Tx2. Two THz pulses are first multiplexed in free-space via a wire-grid polarizer (WGP1), while mutual temporal coherence at the FWWG input is controlled by interposing two silica wedges (SWs) in the Tx1 arm. The orientation of WGP1 is set to 0°, resulting in the maximum transmission for Tx1 and maximum reflection for Tx2. The multiplexed THz pulses are then focused and coupled onto the 10 cm-long FWWG. After propagating through the waveguide, the two multiplexed THz pulses are guided towards the detection stage. Demultiplexing and detection are implemented by using another wire-grid polarizer (WGP2) and a standard electro-optic sampling technique in a ZnTe crystal, respectively. When the orientation of the WGP2 is set to 0°, the ZnTe crystal axis is rotated in such a way that only the *x*-polarized signal is recorded (configuration Rx1); by changing the WGP2 to the 90° position, and rotating the ZnTe crystal axis accordingly, only the *y*-polarized signal can be reconstructed (configuration Rx2) (TPX: THz TPX lens; BS: beam splitter; M: mirror; WP: quarter-wave plate; PM: parabolic mirror).

**Fig. 4:**
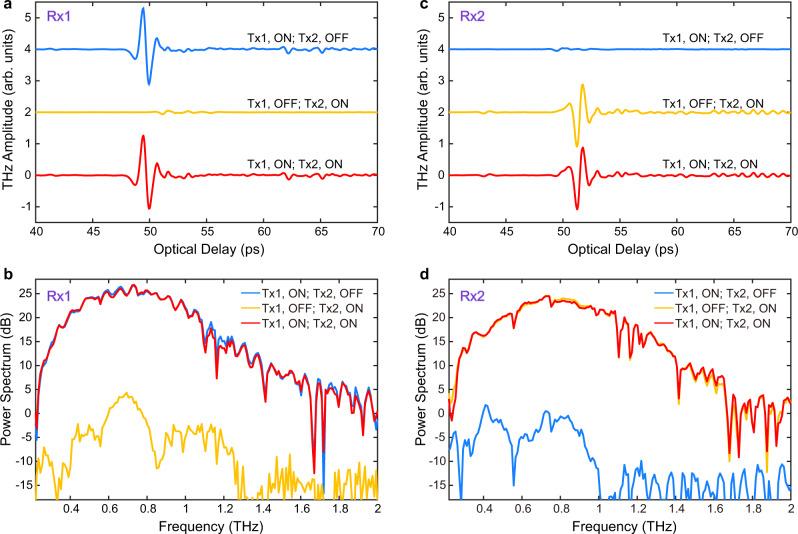
Experimental demonstration of the independent propagation of two polarization-division multiplexed broadband THz pulses within the four-wire waveguide (FWWG). Time-domain signals (**a**) and corresponding spectra (**b**) reconstructed via Rx1 under different ON/OFF configurations of Tx1 and Tx2. Time-domain signals (**c**) and corresponding spectra (**d**) reconstructed via Rx2 under different ON/OFF configurations of Tx1 and Tx2. The THz time-domain waveforms in (**a**) and (**c**) are vertically shifted for clarity.

In particular, based on its two fundamental TEM modes, the FWWG is able to support the independent propagation of two THz pulses multiplexed along the two different polarization axes. For the experimental demonstration, we employ a customized THz time-domain spectroscopy (TDS) system (Fig. 3) with two transmitters (Tx1 and Tx2, generating the *x*- and *y*-polarized beams, respectively) and a single receiver (configured as Rx1 or Rx2 depending on the selected polarization direction). We compare the signals detected via Rx1 and Rx2, under different ON/OFF configurations of Tx1 and Tx2, as shown in Fig. 4. When Tx1 is ON and Tx2 is OFF, the signal from Tx1 is detected via Rx1 and exhibits a single-cycle shape, indicating that the propagation in the FWWG is nearly dispersion-less; when Tx1 is OFF but Tx2 is ON, no significant signal from Tx2 is detected via Rx1, and the corresponding spectra demonstrate an extinction ratio exceeding 20 dB. Concerning the scheme adopted for polarization-division multiplexing, when both Tx1 and Tx2 are ON, the overall signal retrieved via Rx1 and its spectrum is almost identical to those observed when only Tx1 is ON, indicating a negligible contribution from Tx2. When the receiver is switched to Rx2, the results obtained under various ON/OFF configurations exhibit a behavior consistent with the Rx1 configuration (see Fig. 4c, d). Our observations demonstrate that the FWWG can provide the low-loss, almost dispersion-free, and independent propagation of two broadband THz pulses with orthogonal polarization states, and as such, it can be operated as a broadband polarization-division multiplexer.

### Independent manipulation of polarization-division multiplexed THz pulses

**Fig. 5:**
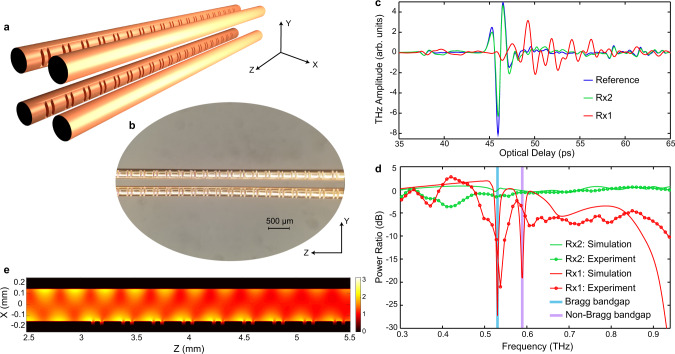
Independent manipulation of polarization-division multiplexed broadband THz pulses within the four-wire waveguide (FWWG). **a** Schematic of the FWWG with Bragg gratings (150-periods-long) engraved along two of the four wires (top and bottom left wires), both placed on the same side of the FWWG. In this case, the multiscale grooves mainly interact with the *x*-polarized THz beam and barely influence the *y*-polarized THz beam. **b** Optical microscopic image of the multiscale grooves engraved along the two wires of the FWWG. **c** Experimental characterization of the FWWG hosting the integrated Bragg gratings, performed by using two broadband THz pulses multiplexed in polarization. The THz signals detected via Rx1 and Rx2 are recorded and plotted together with the reference signal obtained from an unetched FWWG (i.e., no gratings on the wires). **d** Comparison between the simulated and experimental transmission spectra of the FWWG containing the integrated Bragg gratings. **e** Simulated distributions of the electric field modulus evaluated at 0.59 THz, for a *x*-polarized THz beam (cross-sectional view at *y* = *r* + *g*/2).

Finally, we integrate the Bragg gratings on metal-wires into the FWWG, in turn allowing us to realize the independent manipulation of two polarization-division multiplexed broadband THz pulses. We fabricate a FWWG with integrated Bragg gratings by engraving the designed multiscale grooves along the two wires on one side of the FWWG (see Fig. 5a). The transmission spectra of the FWWG with Bragg gratings engraved on different wires are investigated in Supplementary Note 3. Since the grooves are cut along the *y*-direction (in turn making them face along the *x*-direction) and they are relatively shallow (only 40 µm in depth), they mainly interact with the *x*-polarized THz beam and barely influence the *y*-polarized THz beam in the FWWG (see Supplementary Note 5). As a result, this device should ideally feature a notch filter at 0.53 THz for the *x*-polarized THz beam, while behaving as an all-pass filter for the *y*-polarized THz beam. We perform the experimental characterization using the system in Fig. 3 when both Tx1 and Tx2 are ON. As shown in Fig. 5c, compared to the reference (i.e., no grooves), the signal received via Rx1 is attenuated in amplitude and delayed in time. In particular, a long-lasting ringing signal is observed corresponding to the strong Bragg resonance in the frequency domain. In contrast, the signal detected via Rx2 displays the same pulse shape, yet with a slightly lower peak than that of the reference, demonstrating the practically negligible influence of the grooves on the *y*-polarized THz beam.

We investigate the transmission spectra of this device, as shown in Fig. 5d. For Rx1, the experimental transmission spectrum clearly exhibits a Bragg resonance at ~0.53 THz with a notch depth exceeding 20 dB as expected. We note that limited by the fabrication precision of periodic grooves, the Bragg resonance frequencies realized on the two different wires may not exhibit exactly the same value. In addition, due to the imperfect control over the tension manually applied onto the wires, a slight, yet unequal stretch of the periods affects both the sub-*λ* and wavelength-scale structures hosted on different wires. As a result, the overlap of two slightly shifted Bragg resonances leads to the formation of a seemingly single resonance with a wider linewidth. This fact may explain why the *Q*-factor of the observed Bragg resonance (which is ~105.2) is smaller than what we predict in our simulations. Moreover, a non-Bragg bandgap occurs at 0.59 THz in the simulated transmission spectrum. In fact, the origin of this non-Bragg bandgap is attributed to a half-wavelength delay^28^ between the THz electric fields propagating along the asymmetric structure. Figure 5e reveals that the electric field propagating on the side containing the grooves is out of phase with respect to the field propagating on the unetched side. Both location and notch depth of the non-Bragg bandgap are affected by the shifts between the grooves on the two wires, where the notch depth reaches a maximum when the grooves are perfectly aligned (see Supplementary Note 4). In the experiments, due to the uneven tightening of the wires, the shifts between the grooves on different wires are non-consistent, as observed in Fig. 4b. This explains why we cannot identify the non-Bragg bandgap, but rather observe an additional ~6 dB loss across the range from 0.6 to 0.9 THz. Nevertheless, these shifts have no impact on the Bragg resonance (see Supplementary Note 4), and thus do not influence the designed filtering function. For Rx2, the experimental transmission spectrum exhibits an all-pass response as predicted in our simulations, except for a slight loss (<5 dB) in the low frequency range. Such scattering loss is due to the existence of cutting burrs, unintentionally created around the groove edges during the fabrication process (see Methods).

To summarize, we have introduced a universal approach for the realization of broadband THz signal processing in metal-wire waveguides by directly engineering the wire surfaces. The concept of engraving grooves with multiscale structures, combining the merits of photonic crystals and metamaterials, offers additional degrees of freedom to tailor the spectral response of the entire structures. This paves the way for the realization of versatile signal-processing functionalities, including custom-engineered filtering^29^, time differentiation and integration^30^, as well as modulation/demodulation. In parallel, we have experimentally demonstrated the FWWG topology, which provides two independent fundamental modes with orthogonal polarization states for the low-loss and nearly dispersion-free propagation of polarization-division multiplexed THz pulses. By judiciously incorporating the multiscale-structured Bragg gratings into the FWWG, we have demonstrated a unique platform, featuring significant potential towards the independent manipulation of signal channels multiplexed in both frequency (due to its inherent broadband nature) and polarization. Such a platform is anticipated to dramatically enhance THz systems’ capacity and eventually achieve data-rates of ~Tb/s in future THz networks. We envision that the proposed platform can be widely applied in novel application scenarios^2,31,32^, such as multi-channel transmission of uncompressed ultra-high-definition videos, ultra-high-speed short-distance data transfer between THz network elements, as well as chip-to-chip communications.

## Methods

### Simulations

Based on a finite-element-method (FEM) approach, the simulation results presented in Fig. 2 were obtained by means of the *COMSOL Multiphysics* mode solver. The frequency dependent relative permittivity $\varepsilon (\omega )$ of copper at THz frequencies were modeled according to the Drude model^33^:

1 $$\varepsilon (\omega )=1-\frac{{\omega }_{p}^{2}}{{\omega }^{2}+{{{{{\rm{i}}}}}}\omega \varGamma }\mathop{\approx }\limits_{THz}-\frac{{\omega }_{p}^{2}}{{\varGamma }^{2}}+{{i}}\frac{{\sigma }_{0}}{{\varepsilon }_{0}\omega };\,{\sigma }_{0}\mathop{\approx }\limits_{{{THz}}}\frac{{\varepsilon }_{0}{\omega }_{p}^{2}}{\varGamma },$$

where ${\varepsilon }_{0}$ is the free-space permittivity, $\omega$ is the angular frequency, ${\omega }_{p}$ is the angular plasma frequency, and $\varGamma$ is the electron scattering rate. In the THz frequency range, the Drude model is especially simple as it assumes a frequency independent real part of the relative permittivity ${\varepsilon }_{r}=-1.7\times {10}^{5}$ and a frequency independent conductivity ${\sigma }_{0}$=5.96 × 10^7^ S/m. Therefore, the frequency-dependent relative permittivity $\varepsilon (\omega )$ of copper at THz frequencies can be modeled as^33^: $\varepsilon (\nu )=-1.7\times {10}^{5}+{{i}}1.1\times {10}^{6}{\nu }^{-1}$, where $\nu =\omega /2\pi$ in THz. Scattering boundary conditions were used in the simulations. The THz response of the sub-*λ* and wavelength-scale periodic grooves in metal-wire waveguides was numerically investigated via finite-difference-time-domain (FDTD) simulations. The numerical results in Figs. 1 and  5 were obtained using the FDTD module of *Lumerical*. A perfect electric conductor was used for the waveguide, whereas 16 standard perfectly matched layers were employed to absorb spurious reflections coming from the domain edges. To excite the waveguide, we used a broadband signal with a center frequency of 0.5 THz and a 1-THz-bandwidth, carried by a Gaussian beam profile focused down to a waist size of 600 µm at the center of the FWWG input gap. The FDTD simulations were evaluated in a time window of 460 ps with a time resolution of 0.02 ps.

### Experiments

The experiments were performed using the setup illustrated in Fig. 3, which is a customized THz-TDS system with two independent broadband transmitters and a single receiver. The system was driven by an ultrafast near-infrared pulse train (800 nm, 120 fs, 80 MHz) generated by a Ti:Sapphire laser oscillator. The pump and probe beams were obtained by means of a 90/10 beam splitter (BS1). The pump beam was further divided by a 50/50 beam splitter (BS2) to excite the two transmitters. Two identical PCAs (iPCA-21-05-300-800-h, BATOP) served as the transmitters. THz pulses were produced by exciting the PCAs with an average optical power of 500 mW. A bipolar square-wave bias voltage, oscillating at 5 kHz and with a peak-to-peak amplitude of 12 V, was generated by a low-noise function generator and applied to the PCAs. A THz TPX lens (BATOP) with a diameter of 25.4 mm and a focal length of 10 mm was used to focus the THz pulses into the waveguide. Such a lens allowed us to focus the THz beam down to a waist diameter of 400 µm at 0.5 THz. The THz pulses were detected by carrying out the electro-optic sampling technique in a 3 mm-thick ZnTe <110> crystal. The use of such a thick crystal allowed us to achieve a larger temporal window in the measurements, which was necessary to acquire the long-lasting THz transient as modulated by the multiscale grooves. Each recorded THz waveform was centered in a time window of 100 ps with a time resolution of 0.05 ps.

### Fabrications

The fabrication process of the grooves on the bare copper wires was performed at the LMN laboratory at the INRS-EMT, Canada. First, the wires were kept as straight and flat as possible and were glued on a quartz plate. Then the quartz plate with the wires was installed on the base of an automatic dicing saw. The thickness of the employed diamond blade was 35 µm, which determines the width of the grooves. The grooves along the wires were fabricated by utilizing the three-dimensional motor control of the dicing saw. Deviations in the depth were mainly induced by the blade wearing over several cuts. The width of the grooves was constant with the exception of a few burrs at the edge of the cuts. To mount the wires^22^, two PMMA slabs with holes were used to hold and support them. The diameter of the holes was set to 1037 µm in order to guarantee that the gap size between wires was 300 µm in each direction. Screws on the slabs were used to apply tension to the wires, so as to maintain a uniform gap all along the waveguide.

## Supplementary information

Supplementary Information

## Data Availability

All experimental raw data that support the findings of this study are provided in the Source Data file. All the other relevant data are available from the corresponding authors upon reasonable request. Source data are provided with this paper.

## Source data

Source Data

## Code availability

Any simulation and computational codes for this study are available from the corresponding authors upon reasonable request.
