# Supplementary material for: Versatile metal-wire waveguides for broadband terahertz signal processing and multiplexing
Source: Nat Commun. 2022 Feb 8;13:741. doi: 10.1038/s41467-022-27993-7 (PMC8826316; doi:10.1038/s41467-022-27993-7)
Supplement: Supplementary file 1 — Supplementary Information [file 41467_2022_27993_MOESM1_ESM.docx]

*Supplementary Information*

Versatile metal-wire waveguides for broadband terahertz signal processing and multiplexing

*Junliang Dong^1,*^, Alessandro Tomasino^1^**, Giacomo Balistreri^1,2^, Pei You^1^, Anton Vorobiov^3^,* *Étienne Charette^1^, Boris Le Drogoff^1^, Mohamed Chaker^1^, Aycan Yurtsever^1^, Salvatore Stivala^2^, Maria A. Vincenti^4^, Costantino De Angelis^4^, Detlef Kip^3^, José Azaña^1^, and Roberto Morandotti^1,*^*

*^1.^Institut national de la recherche scientifique, Centre Énergie Matériaux Télécommunications, Varennes, QC J3X 1P7, Canada*

*^2.^Department of Engineering, University of Palermo, Viale delle Scienze, Palermo 90128, Italy*

*^3.^Faculty of Electrical Engineering, Helmut Schmidt University, Holstenhofweg 85, Hamburg 22043, Germany*

*^4.^Department of Information Engineering, University of Brescia, Via Branze 38, Brescia 25123, Italy*

*^*^Corresponding authors: J.D.:* [*Junliang.Dong@inrs.ca*](mailto:Junliang.Dong@inrs.ca) *and R.M.:* [*Roberto.Morandotti@inrs.ca*](mailto:Roberto.Morandotti@inrs.ca)

***Supplementary Note 1:*** *Propagation characteristics of THz spoof surface plasmon polaritons along metal-wires hosting multiscale grooves*

In the THz frequency regime, metals are generally considered as perfect conductors, as the negligible penetration of the electromagnetic field leads to highly delocalized surface plasmon polaritons (SPPs) akin to grazing-incidence light fields. The propagation characteristics of THz SPPs along the metal-air interface can be described by their dispersion relation, i.e., a map between the angular frequency *ω* and the propagation constant *β*(*ω*) of the fundamental mode. When periodic structures are engraved on the metal, surface waves resembling the behavior of SPPs, namely spoof SPPs^1^, can be still sustained, and their dispersion relation can be tailored by varying the geometry of the periodic structures. For a metal-insulator-metal plasmonic waveguide structure with periodic grooves engraved on both metallic surfaces (counter-facing towards each other), so-called a spoof-insulator-spoof (SIS) waveguide structure^2^, the analytical expression of its dispersion relation is given by^3^:

$\beta=k_{0}\sqrt{1+\frac{2w}{pgk_{0}}\tan\left(k_{0}d\right)}$, (1)

where $k_{0}=\omega/c$ denotes the propagation constant of plane waves in free space, *c* being the speed of light. The width *w*, depth *d*, and period *p* describe the geometry of the grooves, where *g* is the size of the air gap that separates the two flat metallic surfaces. When the duty cycle *w/p* is equal to 0 (or the depth *d* is 0), this condition indicates that there are no grooves etched on the metal and thus, the dispersion relation has an exact linear dependence on the frequency (i.e., no dispersion). When the geometry of the grooves is in the subwavelength scale, the effective medium theory can be applied^4^, thus the effective refractive index *n_eff_* is expressed as^3^:

$n_{eff}\equiv\frac{\beta}{k_{0}}=\sqrt{1+\frac{2w}{pgk_{0}}\tan\left(k_{0}d\right)}$. (2)

Based on Supplementary Equations 1 and 2, it is clear that the propagation characteristics of the spoof SPPs can be easily tuned by engineering the depth of the grooves *d*, the duty cycle *w/p*, as well as the gap size *g* between the two metal surfaces.

In principle, the THz guidance in metal-wire waveguides also relies on the propagation of SPPs along the metal-air interface^5^. In our study, we aim at tailoring the propagation characteristics of the SPPs confined in-between two metal-wires by corrugating the wire surfaces with periodic grooves, as shown in Supplementary Figure 1(a). Although such a structure can be also considered as a SIS waveguide, its dispersion relation cannot be directly expressed using Supplementary Equation 1. This is because Supplementary Equation 1 can only be used to describe the propagation characteristics of the spoof SPPs confined in-between two plane conductors. Due to the non-planar surface of the metal-wires, the depth of the grooves *d* and the air gap between the two wires *g* are not constant along the cutting direction (*y*-direction), in turn making it extremely difficult to derive an analytical expression of the dispersion relation. In order to achieve the most accurate results, finite-difference-time-domain (FDTD) simulations were performed. The dispersion relation shown in Fig. 1(c) (in the main manuscript) describes the propagation characteristics of the spoof SPPs for a depth *d* = 40 µm, a width *w* = 35 µm, and a period *p* = 80 µm. The dispersion relation shows a cut-off frequency *f_c_* at ~1.2 THz, demonstrating that the spoof SPPs at such a frequency are stopped (propagation velocity equals to 0) and the THz frequencies above *f_c_* cannot be guided within the waveguide. By adjusting the geometry of the grooves to the wavelength scale, the cut-off frequency *f_c_* can be accordingly shifted to the lower frequency range, in turn narrowing the operating bandwidth. Therefore, we could expect that, in principle, it is possible to modify the propagation velocity and the cut-off frequency of the propagating spoof SPPs, by tailoring the geometry of the grooves, where we change the depth *d* (or duty cycle *w/p*) while keeping the periodicity; however, with such a procedure, a Bragg resonance cannot be achieved within the operating THz bandwidth, in contrast to what is typically done in optical waveguides.

***
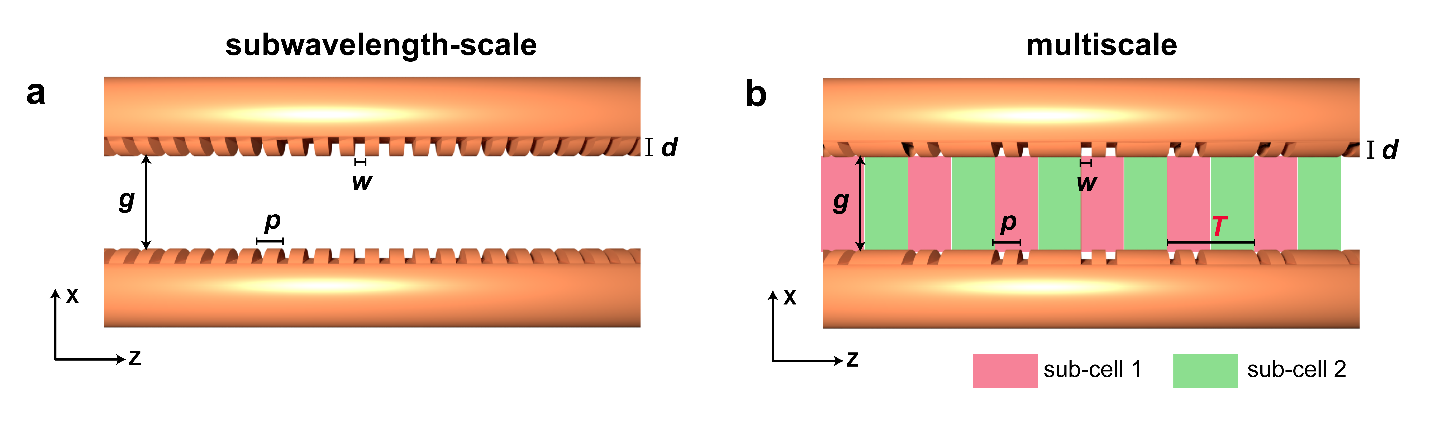
***

**Supplementary Figure 1** (**a**). Schematics of the TWWG with subwavelength-scale periodic grooves. The grooves are engraved on both wires and face each other. (**b**). Schematics of the TWWG with multiscale grooves.

In order to overcome this issue and introduce a Bragg resonance without influencing the bandwidth, we introduce, both theoretically and experimentally, the concept of multiscale structures into the THz regime. A multiscale structure is achieved by superimposing a wavelength-scale periodic modulation *T* onto the subwavelength-scale periodic grooves. Such a multiscale structure can be interpreted as the combination of two sub-cells with different propagation constants *β_i_*, where the subscripts *i=1*,*2* correspond to each individual sub-cell. Any geometrical difference (depending on *d* and *w/p*) between the sub-cells results in different *n_eff_*, in turn leading to a periodic modulation at the wavelength scale, as shown in Supplementary Figure 1(b). The dispersion relation *β_m_* of the spoof SPPs propagating along such a multiscale structure can be obtained from the Bloch theorem^6^:

$\cos\left[\beta_{m}\left(p_{1}+p_{2}\right)\right]=\cos\left(\beta_{1}p_{1}\right)\cos\left(\beta_{2}p_{2}\right)-\frac{1}{2}\left(\frac{\beta_{1}}{\beta_{2}}+\frac{\beta_{2}}{\beta_{1}}\right)\sin\left(\beta_{1}p_{1}\right)\sin\left(\beta_{2}p_{2}\right)$ (3)

In our design shown in Supplementary Figure 1(b), we have *p_1_=80* µm, *p_2_=60* µm and *T=2p_1_+2p_2_=*280 µm*.* In particular, for the sub-cell 2, the duty cycle *w/p* equals to 0 (or *d=0*). The solution of Supplementary Equation 3 exhibits a bandgap at the irreducible Brillouin zone boundary^3^, where *β_m_=π/T.* In this condition, the upper limit of the bandgap edge occurs at *f_bandgap_*= *β_m_c/2π=c/(2T)*, which also satisfies the Bragg condition. The simulated dispersion relation in Fig. 1(c) (in the main manuscript) confirms the existence of a Bragg bandgap at 0.53 THz. By simply altering the period of the wavelength-scale modulation *T*, the location of such a Bragg bandgap can be tuned within a bandwidth as large as ~1 THz. Based on Supplementary Equation 3, it is clear that the concept of multiscale structures offers more degrees of freedom to tailor the spectral response of the entire structure and, as such, it is considered as an effective tool for manipulating the properties of the spoof SPPs that propagate in metal-wire waveguides.

***Supplementary Note 2:***  *Signal-transporting capability of the FWWG*

**
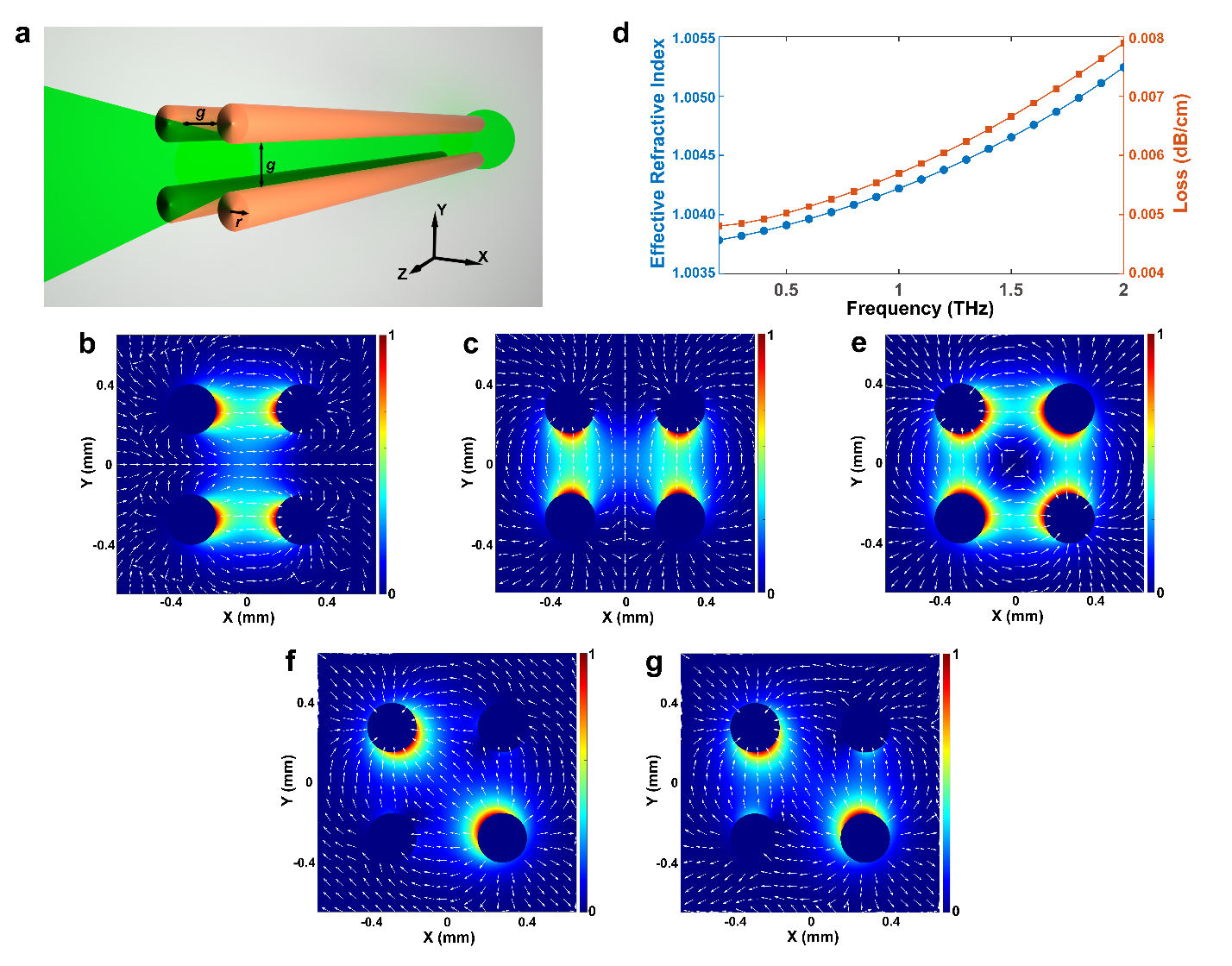
**

**Supplementary Figure 2** THz guiding properties of the FWWG. (**a**) Schematic of the FWWG. A THz beam is coupled into the waveguide comprised of four identical copper wires with an equal separation *g* = 300 µm. The radius *r* of the copper wires is 127 µm. (**b, c**) FEM simulations of the normalized electric field intensity distributions of the fundamental TEM modes evaluated at 0.5 THz, TEM_x_ (**b**) and TEM_y_ (**c**), which can be efficiently excited by *x*- and *y*-polarized THz beams, respectively. (**d**) Simulated effective refractive indices and absorption losses of the fundamental modes as a function of frequency. (**e**) Normalized electric field intensity distribution of the quadrupole mode. (**f, g**) Simulated normalized electric field intensity distributions at 0.5 THz when the incident THz beam features polarization angles of 135˚ (**f**) and 150˚ (**g**), respectively. The arrows in the 2D distributions indicate the local electric field polarization directions.

The THz guiding properties of the four-wire waveguide (FWWG, as depicted in Supplementary Figure 2(a)) are investigated by numerical simulations based on a finite-element-method (FEM) approach. The simulated fundamental TEM modes of the FWWG, evaluated at 0.5 THz, are shown in Supplementary Figures 2(b) and 2(c). Due to the symmetric arrangement of the four wires, the two fundamental modes, TEM_x_ and TEM_y_, exhibit symmetric field profiles, which are equally divided into two identical portions along the axes. Notably, each portion of the field distribution is mainly confined in-between the two corresponding wires and shows a similar profile to that of a single two-wire waveguide, thus indicating that the FWWG can also be efficiently excited by a linearly-polarized THz beam. In details, TEM*_x_* is excited within the FWWG by a *x*-polarized THz beam, while TEM*_y_* is excited by a *y*-polarized THz beam. Based on the properties exhibited by THz metal-wire waveguides^7^, the effective refractive index *n_eff_* of both fundamental modes is close to 1, while the associated losses are particularly low (< 0.008 dB/cm), as retrieved from simulations and shown in Supplementary Figure 2(d). These results prove that the FWWG can provide the low-loss and almost dispersion-less propagation of broadband THz signals. In addition, we note that, besides the two symmetric fundamental modes, a higher-order mode, namely a quadrupole, is also identified in the mode analysis, as shown in Supplementary Figure 2(e). However, the polarization status of this mode is not symmetric along either the *x*- or *y*-axis, indicating that such a quadrupole mode cannot be excited within the FWWG via a linearly-polarized THz beam.

A unique feature of the FWWG is the capability to guide linearly polarized THz pulses with arbitrary polarization direction. For example, in Supplementary Figures 2(f) and S2(g), we show the normalized electric field intensity distributions within the FWWG when the polarization angles of the input THz beams are 135˚ and 150˚, respectively. To analyze the field distributions and their relationships with the fundamental modes, the charge weight^8^ of the fundamental modes is calculated as:

$C_{i}=\frac{\iint_{\infty} e(x,y)\cdot\psi_{i}^{*}(x,y)dxdy}{\left(\iint_{\infty} e(x,y)\cdot e^{*}(x,y)\iint_{\infty} \psi_{i}(x,y)\cdot\psi_{i}^{*}(x,y)\right)^{1/2}}$ , (S4)

where $C_{i}$ is the *i*-th charge weight, which satisfies the normalization condition $\sum\left| C_{i} \right|^{2}=1$. Here *e*(*x,y*) is the electric field transverse profile across the FWWG input section and *ψ_i_*(*x,y*) is the electric field distribution of the fundamental mode, corresponding to either TEM*_x_* or TEM*_y_*. For the case in Supplementary Figure 2(f), the charge weights are $C_{x}=-1/\sqrt{2}$ and $C_{y}=1/\sqrt{2}$. Likewise, the charge weights are $C_{x}=-1/2$ and $C_{y}=\sqrt{3}/2$ for the case in Supplementary Figure 2(g). It is evident that the charge weights are determined by the polarization directions, while the electric field distribution within the FWWG is given by the weighted superposition of the two fundamental modes. This observation indicates that a THz beam coupled into the FWWG with arbitrary polarization directions is decomposed into the two orthogonal polarization states, the fundamental TEM modes of which propagate independently.

We perform the experimental demonstration based on the THz-TDS system shown in Fig. 3 (in the main manuscript). Since only one transmitter is needed for this experiment, Tx2 is de-activated and the two wire-grid polarizers are removed. The control of the polarization direction of the input THz beam is realized by rotating the PCA in transmitter Tx1. Accordingly, the ZnTe detection crystal is rotated in order to be sensitive to the THz signal with a specific polarization direction. The experimentally detected time-domain signals and the corresponding spectra, calculate via a Fast Fourier Transform (FFT) algorithm, are shown in Supplementary Figures 3(a) and 3(b). By changing the polarization directions of the input THz beams, the detected signals feature, after propagating through the 10-cm-long FWWG, the same shape in both the time-domain waveforms and the frequency spectra. A slight difference in the low-frequency region of the spectra is most likely due to a small misalignment of the THz beam with respect to the waveguide input, induced by the rotation of the transmitter. Based on the recorded experimental results, we can conclude that the FWWG can transport broadband THz pulses with arbitrary polarization directions in a low-loss and low-dispersion manner. It is worth mentioning that, in contrast to all the other existing THz waveguides^9^, this is a unique characteristic of the FWWG.


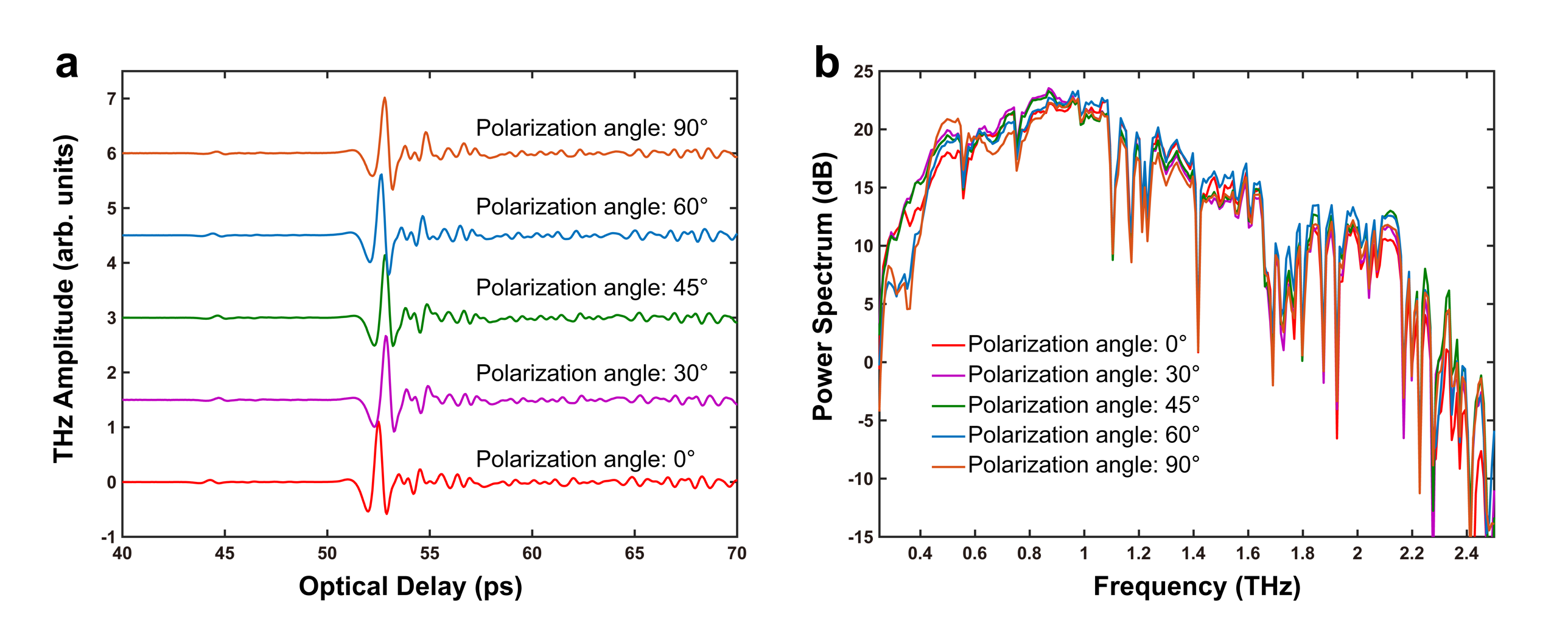


**Supplementary Figure 3** FWWG response as a function of the polarization of the input broadband THz pulse. Experimentally reconstructed time-domain signals at the output (**a**) and corresponding FFT-calculated spectra (**b**) when the polarization directions of the input THz beams are 0˚ (purely *x*-polarized), 30˚, 45˚, 60˚, and 90˚ (purely *y*-polarized), respectively (indicated in the plots). The THz waveforms in (**a**) are vertically shifted for clarity.

***Supplementary Note 3:*** *Transmission spectra of the FWWG with Bragg gratings engraved on different wires*

In principle, in order to achieve the most effective notch-filtering of the *x*-polarized THz signals, the designed 150-period multiscale grooves should be engraved on all the four wires by cutting them along the *y*-direction, leading to the grooves facing each other in the *x*-direction. However, in the experiments, achieving a perfect manual alignment of the grooves among all four wires is extremely challenging. To minimize the potential misalignment, only two wires of the FWWG are engraved with the multiscale grooves. The proper choice of wires is investigated via FDTD simulations. We note that all the simulated transmission spectra plotted in the Supplementary material are achieved by calculating the ratio between the transmitted power spectrum of the FWWG hosting the designed structures and that of a plain FWWG (with no grooves). As shown in Supplementary Figure 4, when all the four wires are engraved, the notch depth of the Bragg bandgap that can be achieved is over 35 dB. When two of the four wires are engraved with the multiscale grooves, the notch depth of the Bragg bandgap is optimal when the two wires containing grooves are on the same side of the FWWG, as shown in the red curve of Supplementary Figure 4. The notch depth achieved in this case is over 25 dB. Therefore, the use of only two engraved wires is a judicious trade-off between a reasonable notch depth and the practical realization of the filtering function.


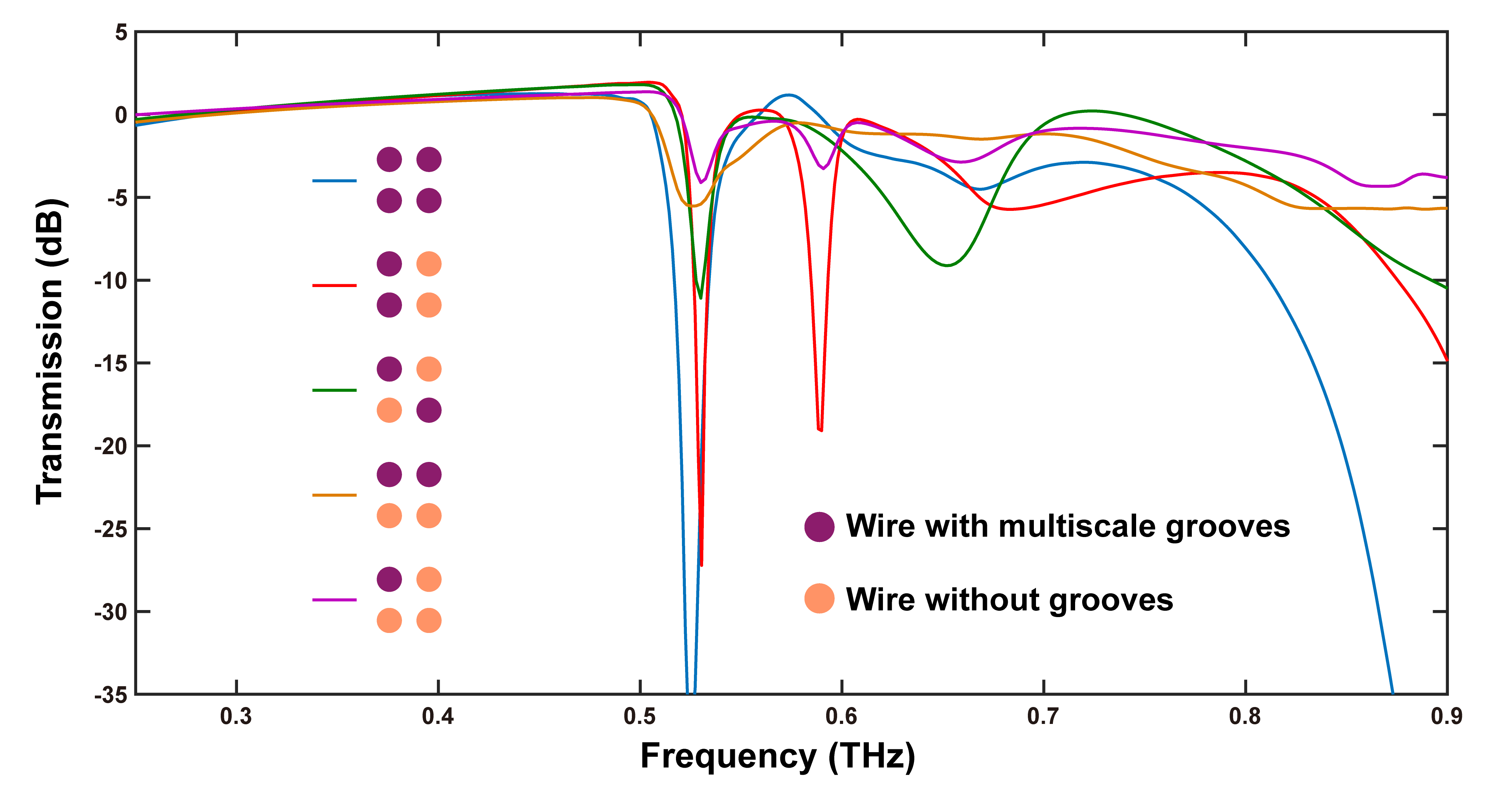


**Supplementary Figure 4** Simulated spectral responses in transmission of the FWWG with 150-period multiscale grooves engraved on different wires, when a *x*-polarized THz beam is sent as the input, considering the cases of perfectly aligned grooves (no lateral shift).

***Supplementary Note 4:*** *Influence of the shifts between grooves in the two wires on the properties of the Bragg and non-Bragg bandgaps*

To investigate the influence that the relative shift between the 150-period multiscale grooves realized on the *two wires* has on the transmission spectrum of the horizontally polarized THz signal, FDTD simulations have been performed. The simulated transmission spectra are shown in Supplementary Figure 5. For the Bragg bandgap, both the location (at 0.53 THz) and the notch depth are not influenced by the shift. In contrast, the notch depth of the non-Bragg bandgap is sensitive to the misalignment between the grooves, and it is maximum when the alignment is perfect^10^, or equivalently, when the grooves shift is in units of the period *T*. The simulated transmission spectra also demonstrate that the shifts mostly affect the frequency range between 0.6 THz and 0.9 THz. It is worth mentioning that the simulated spectra only undertake shifts of the fixed values reported in Supplementary Figure 5. In practice, consistent shifts cannot be guaranteed due to the difference in the tightening of the wires. This may also explain why the non-Bragg stopband is not clearly observed in the experimental transmission spectra.


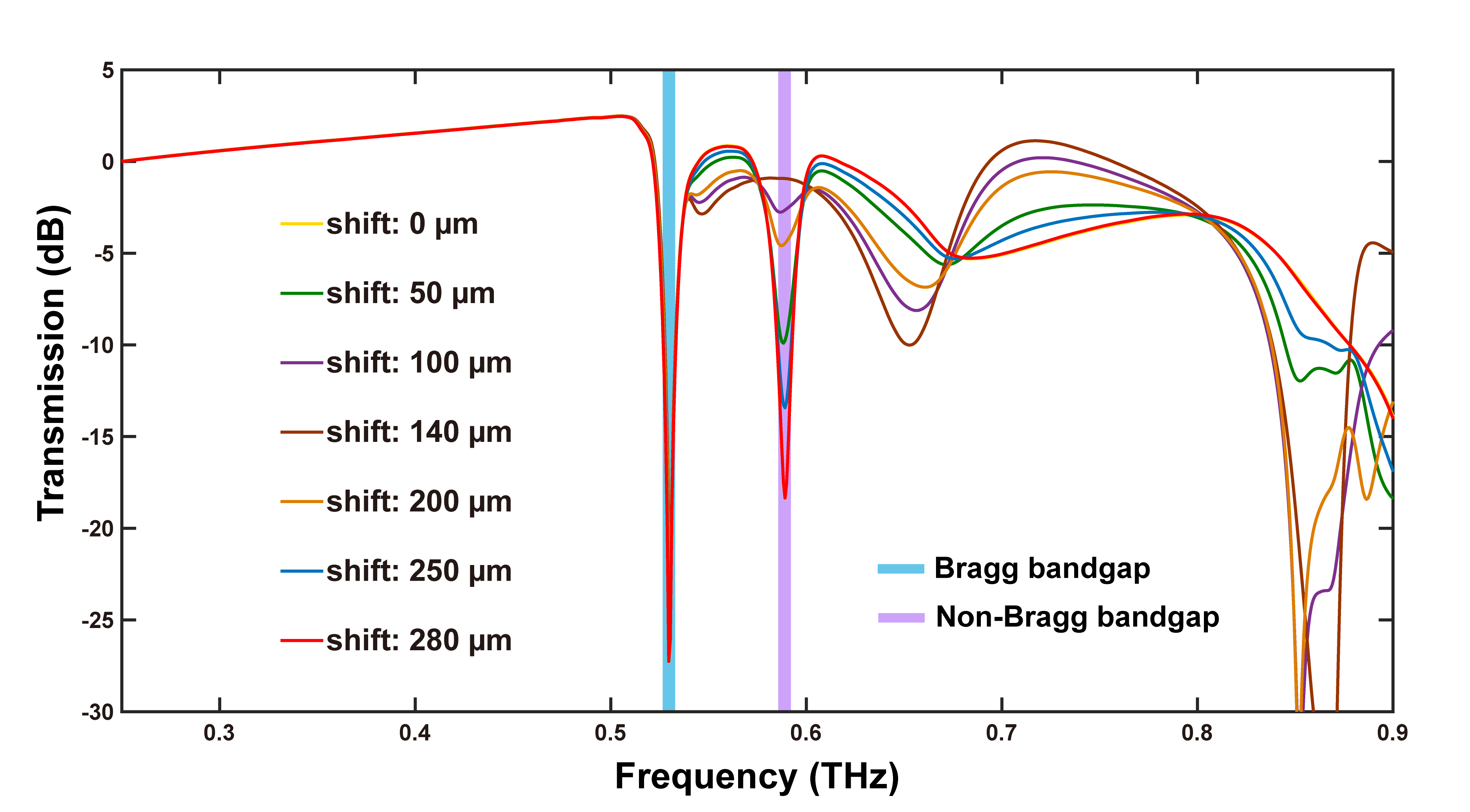


**Supplementary Figure 5** Simulated transmission spectra of the FWWG when the relative shifts between the grooves on the two wires are 0 µm, 50 µm, 100 µm, 140 µm, 200 µm, 250 µm, and 280 µm, respectively.

***Supplementary Note 5:*** *Influence of the depth of the grooves facing along the x-direction on the y-polarized THz beam*

In our design shown in Fig. 5 (in the main manuscript), when the multiscale-structured grooves are cut along the *y*-direction (in turn facing the *x*-direction), they barely influence the *y*-polarized THz mode in the FWWG because they are relatively shallow (only 40 µm in depth). The fact that the grooves are fabricated by engraving only a rather superficial portion of the wire, implies that they affect a perimeter shorter than half of the wire circumference. Because of this, and due to the specific field distribution of the *y*-polarized mode, the grooves are expected to have little effect on this polarization, in a quite large range of depth values. In order to further investigate the influence of groove depth on the *y*-polarized THz mode, FDTD simulations have been performed. The achieved transmission spectra with different groove depths (while the other parameters are kept the same as those in Fig. 5 (in the main manuscript)) are shown in Supplementary Figure 6. Based on the simulation results, we find out that, as the grooves become deeper, the cut-off frequency associated to the *y*-polarized THz mode shifts towards the lower-frequency range. This effect can be explained by considering that the grooves behave as a cavity, which traps the energy associated to increasingly longer wavelengths (i.e. lower frequencies) as their depth get larger. In particular, since the grooves are not oriented towards the *y*-direction, there is no appreciable effective wavelength-scale modulation applied onto the *y*-polarized THz mode, in turn leading to no observation of the Bragg resonance at 0.53 THz. Grooves with depth larger than 80 µm will further decrease the operating bandwidth of the *y*-polarized THz mode, while severely compromising the robustness of the wires. The fact that, at certain frequencies, the transmission exceeds 0 dB is due to the field enhancement induced by the subwavelength-scale grooves.


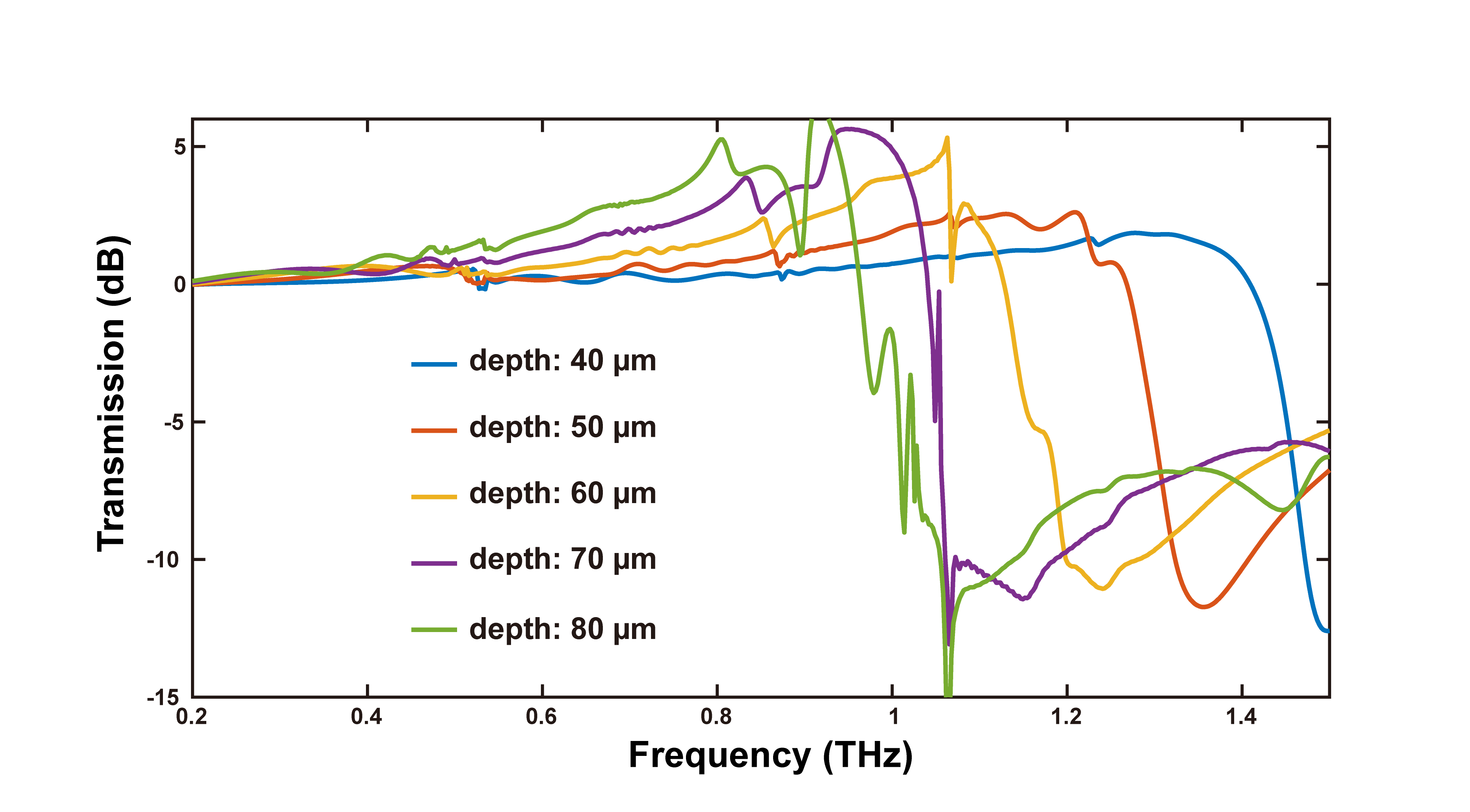


**Supplementary Figure 6** Simulated transmission spectra of *y*-polarized THz beam in the FWWG when the depths of the grooves engraved along the *y*-direction (grooves facing the *x*-direction) are 40 µm, 50 µm, 60 µm, 70 µm, and 80 µm, respectively.

**Supplementary References**

1. Pendry, J. B. Mimicking Surface Plasmons with Structured Surfaces. *Science (80-. ).* **305**, 847–848 (2004).

2. Kats, M. A., Woolf, D., Blanchard, R., Yu, N. & Capasso, F. Spoof plasmon analogue of metal-insulator-metal waveguides. *Opt. Express* **19**, 14860 (2011).

3. Zhang, Q. *et al.* Microwave band gap and cavity mode in spoof-insulator-spoof waveguide with multiscale structured surface. *J. Phys. D. Appl. Phys.* **48**, (2015).

4. Gao, Z., Wu, L., Gao, F., Luo, Y. & Zhang, B. Spoof Plasmonics: From Metamaterial Concept to Topological Description. *Adv. Mater.* **30**, 1–30 (2018).

5. Maier, S. A., Andrews, S. R., Martín-Moreno, L. & García-Vidal, F. J. Terahertz surface plasmon-polariton propagation and focusing on periodically corrugated metal wires. *Phys. Rev. Lett.* **97**, 1–4 (2006).

6. Zhukovsky, S. V., Orlov, A. A., Babicheva, V. E., Lavrinenko, A. V. & Sipe, J. E. Photonic-band-gap engineering for volume plasmon polaritons in multiscale multilayer hyperbolic metamaterials. *Phys. Rev. A* **90**, 013801 (2014).

7. Markov, A., Guerboukha, H. & Skorobogatiy, M. Hybrid metal wire–dielectric terahertz waveguides: challenges and opportunities [Invited]. *J. Opt. Soc. Am. B* **31**, 2587 (2014).

8. Chen, S. & Wang, J. Theoretical analyses on orbital angular momentum modes in conventional graded-index multimode fibre. *Sci. Rep.* **7**, 3990 (2017).

9. Atakaramians, S., Afshar V., S., Monro, T. M. & Abbott, D. Terahertz dielectric waveguides. *Adv. Opt. Photonics* **5**, 169 (2013).

10. Bazargani, H. P., Burla, M., Chrostowski, L. & Azaña, J. Photonic Hilbert transformers based on laterally apodized integrated waveguide Bragg gratings on a SOI wafer. *Opt. Lett.* **41**, 5039 (2016).
